# Properties of silver nanostructure-coated PTFE and its biocompatibility

**DOI:** 10.1186/1556-276X-8-388

**Published:** 2013-09-17

**Authors:** Jakub Siegel, Markéta Polívková, Nikola Slepičková Kasálková, Zdeňka Kolská, Václav Švorčík

**Affiliations:** 1 Department of Solid State Engineering, Institute of Chemical Technology, Prague 166 28, Czech Republic; 2 Faculty of Science, J.E. Purkyně University, Usti nad Labem 400 96, Czech Republic

**Keywords:** Deposition, Silver nanostructure, Thermal treatment, Surface characterization, Cell adhesion and proliferation

## Abstract

Silver nanolayers were sputtered on polytetrafluoroethylene (PTFE) and subsequently transformed into discrete nanoislands by thermal annealing. The Ag/PTFE composites prepared under different conditions were characterized by several complementary methods (goniometry, UV-visible spectroscopy, X-ray photoelectron spectroscopy, and atomic force microscopy), and new data on the mechanism of Ag layer growth and Ag atom clustering under annealing were obtained. Biocompatibility of selected Ag/PTFE composites was studied *in vitro* using vascular smooth muscle cell (VSMC) cultures. Despite of the well-known inhibitory properties of silver nanostructures towards broad spectrum of bacterial strains and cells, it was found that very thin silver coating stimulates both adhesion and proliferation of VSMCs.

## Background

Nanomaterials and nanoparticles have recently received considerable attention because of their unique properties and diverse applications in biotechnology and life science. Nanosilver products, which have well-known antimicrobial properties, have been used extensively in a range of medical settings [1-5].

Bactericidal properties of silver in the form of ions, nanoparticles, or composite nanodevices based on thin Ag films have been broadly reported [6,7]. Antibacterial properties, however, are one, but not the only prerequisites for successful integration of functional artificial materials into living tissues. Biocompatibility and side cytotoxicity of such materials have to be considered too. Cell survival and cell death are two major toxicity endpoints that can be rapidly and effectively measured using *in vitro* experimental models employing cultured mammalian cells [8-10].

Antibacterial surface modification of biomedical materials has evolved as a potentially effective method of preventing bacterial proliferation and biofilm formation on medical devices [11]. Microbial colonization and biofilm formation on implanted devices represent an important complication in, e.g., orthopedic surgery, dental surgery, or during replacement of skin cover after severe post-traumatic conditions (burns and abrasions), and may result in implant failure. Controlled release of antibacterial agents directly at the implant site may represent an effective approach to treat these chronic complications [9]. Recent advances in the field of nanotechnology led scientific opinion to consider metal nanoparticle recruitment a very promising tool to fight antibiotic-resistant bacteria [10,11]. Among the nanomaterials, silver nanoparticles (AgNPs) have shown good inhibitory and antimicrobial efficacy against a significant number of pathogens (such as bacteria, viruses, yeasts, and fungal species) [12], without provoking microbial resistance [13]. Moreover, silver ions have demonstrated the capability to inhibit biofilm formation [14]. Resistance to conventional antibiotics by pathogenic bacteria has emerged in recent years as a major problem of public health. In order to overcome this problem, non-conventional antimicrobial agents have been under investigation. Silver-based medical products, ranging from bandages for wound healing to coated stents and catheters, have been proved effective in retarding and preventing infections of a broad spectrum of bacteria [15]. Surface proteins are probably the most Ag^+^-sensitive sites, and their alterations result in bacterial disruption due to structural and severe metabolic damage. Silver ions inhibit a number of enzymatic activities by reacting with electron donor groups, especially sulfhydryl groups [16]. In contrast to the antibacterial properties of silver (both as ions and as metallic nanoparticles), its potential cytotoxic effects on eukaryotes have not yet been satisfactorily elucidated [17]. However, it is clear that the potential adverse effects of AgNPs issued from their ability to penetrate the membrane and then interfere with various metabolic pathways of the cell [18]. Improvements in the development of non-cytotoxic, bactericidal silver-containing products are therefore being continuously sought. In particular, increasing interest is being shown towards the safe exploitation of silver nanotechnology in the fabrication of bioactive biomaterials.

The main aim of this paper is to find out whether the silver nanostructures, which are generally known for their inhibitory properties towards broad spectrum of bacterial strains, deposited on polytetraethylfluorene (PTFE) conform to cell cultures cultivated on this composite. For this purpose, silver-coated PTFE samples are prepared; their properties, which are expected to affect the interaction with cells, are characterized by different complementary experimental techniques. Special emphasis is paid to the effects of surface morphology, chemical composition, and amount of silver. Biological activity of silver-coated PTFE is examined *in vitro* on vascular smooth muscle cells (VSMCs).

## Methods

### Materials, Ag deposition, and thermal treatment

PTFE foil (thickness 50 μm, density 2.2 g cm^−3^, melting temperature *T*_m_ = 327°C), supplied by Goodfellow Cambridge Ltd. (Huntingdon, UK), was used for this experiment. The PTFE samples were silver coated by diode sputtering using Balzers SCD 050 device (Goodfellow Ltd.). The deposition of silver was accomplished from Ag target (purity 99.99%), supplied by Safina a.s. (Czech Republic). The parameters of the deposition were as follows: DC Ar plasma, gas purity 99.995%, gas flow 0.3 l s^−1^, pressure 5 Pa, power 20 mA, inter-electrode distance 50 mm, and sputtering time varied from 10 to 200 s. The thermal annealing was performed immediately after Ag deposition on air at 250°C for 1 h using thermostat Binder oven (Tuttlingen, Germany). The annealed samples were cooled down on air to room temperature. The experiments were performed on the samples of pristine PTFE, the Ag-coated PTFE, immediately after the Ag deposition (as-sputtered) and after 14 days from the deposition (relaxed). The annealed samples, relaxed for 14 days from the annealing (annealed), were used in further experiments.

### Measurement techniques

Surface wettability was characterized by contact angle (CA) measured by goniometry using static water drop method. The analysis was performed at ten different positions (room temperature) using distilled water (volume of water drop was 8 μl ± 0.2 μl). The evaluation of the contact angles was performed by a three-point method using software SeeSystem 6.3 (Advex Instruments s.r.o., Brno, Czech Republic).

UV-visible spectroscopy (UV–vis) absorption spectra were measured using Perkin Elmer UV/VIS Spectrometer Lambda 25 (Waltham, MA, USA) in the spectral range of 300 to 800 nm with recording rate of 240 nm s^−1^.

The atomic concentrations of Ag (3*d*), O (1 *s*), F (1 *s*), and C (1 *s*) in Ag-coated (as-sputtered, relaxed, and annealed) PTFE were determined by X-ray photoelectron spectroscopy (XPS) method on Omicron NanotechnologyESCAProbeP spectrometer (Omicron NanoTechnology GmbH, Taunusstein, Germany). The analyzed area had a dimension of 2 × 3 mm^2^. The X-ray source was monochromated at 1,486.7 eV, and the measurement was performed with a step size of 0.05 eV. The spectra evaluation was carried out using CasaXPS software (Tel Aviv, Israel).

The surface morphology and roughness of pristine, relaxed, and annealed PTFE samples Ag coated for different deposition times were examined by atomic force microscopy (AFM) using VEECO CP II device working in tapping mode. A phosphorous-doped silicon probe RTESPA-CP (Veeco, Mannheim, Germany) with a spring constant of 20 to 80 N m^−1^ was chosen. The mean roughness value (*R*_*a*_) represents the arithmetic average of the deviation from the center plane of the sample.

### Cell colonization

The interaction of pristine and Ag-coated PTFE surface (relaxed and annealed) with the cell was studied by *in vitro* method. The VSMCs from the rat aorta were used in this experiment. For the studies of cell adhesion and proliferation, the pristine and Ag-coated (sputtering times 20, 100, and 200 s) PTFE was chosen. The samples were sterilized for 1 h in ethanol (75%) and air dried before the experiment. The samples were inserted into 12-well plates (TPP, Trasadingen, Switzerland) and seeded with VSMCs with the density of 17,000 cells cm^−2^ into 3 ml of Dulbecoo’s modified Eagle’s essential medium (Sigma, USA) supplemented with 10% fetal bovine serum (Sebak GmbH, Germany). The cells were cultured for 1, 2, 5, and 7 days at 37°C in a humidified atmosphere containing 5% CO_2_.

For cell number analysis and cell distribution on sample surface, the method of randomly chosen fields was chosen. On the first, second, fifth, and seventh day from seeding, the cells were rinsed with phosphate-buffered saline (Sigma), fixed for 45 min in 75% cold ethanol (at 20°C), and stained (1 h) with a combination of the fluorescence dyes. Texas Red C_2_-maleimide (Invitrogen Ltd., Renfrew, UK) was used for dying the cell membrane. The cell nuclei were visualized using Hoechst #33342 (Sigma). The fluorescent microscope Olympus IX-51 (Evropská, Czech Republic) with digital camera DP-70 was used for the creation of the 20 photographs from different positions of the samples. The number of cells was determined using NIS-Elements AR3.0 software (Nikon, Melville, NY).

## Results and discussion

Since the cell adhesion and proliferation are strongly affected by chemical composition, surface morphology, wettability, and other physicochemical properties of underlying carrier, the silver/PTFE composites prepared under different conditions were characterized by various complementary analytical methods.

### Contact angle measurement

**Figure 1:**
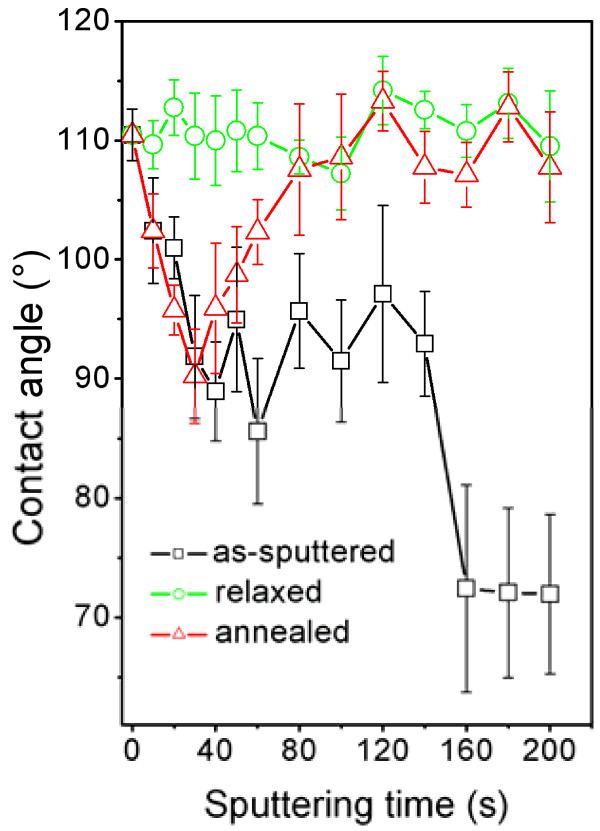
**Dependence of contact angle on sputtering time for pristine (deposition time 0 s) and silver-coated PTFE.** Contact angle was determined immediately after Ag deposition (as-sputtered), after 14 days from the Ag deposition (relaxed), and on annealed and relaxed samples (annealed).

The dependence of the CA of silver-coated PTFE on the silver sputtering time from 10 to 200 s is shown in Figure 1 and compared with that of pristine PTFE (CA = 110.5° ± 2.0°). The contact angle was determined immediately after silver deposition (as-deposited), after 14 days from the silver deposition (relaxed), and on annealed and relaxed samples (annealed).

**Figure 2:**
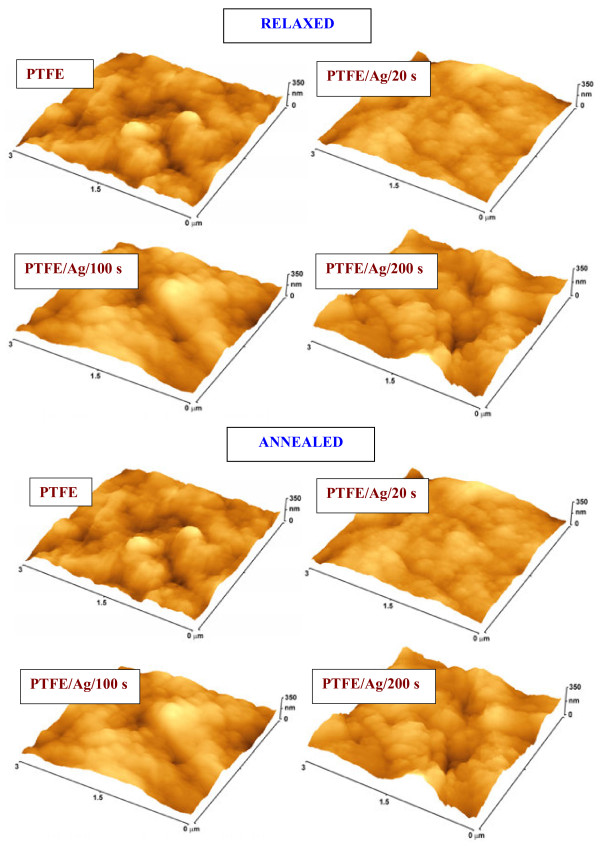
**AFM images.** AFM images of pristine and Ag-coated PTFE (20, 100, and 200 s) for relaxed and annealed samples.

The deposition of Ag layer onto PTFE results in significant CA decrease (i.e., increase of wettability), due to pronounced masking effect of the Ag layer. This decrease is most pronounced in the case of the thickest Ag coatings (sputtering time > 160 s), for which the creation of fully continuous coverage is expected in accordance with previous work [19]. For the as-deposited samples, three distinguishable regions are seen on the dependence of CA on the sputtering time. In the first region, the contact angle is a decreasing function of sputtering time (deposition time 10 to 40 s). The second region is characterized by nearly constant, within experimental error, CA value of about 92° (sputtering times 40 to 140 s). In the third region (sputtering time > 160 s), the contact angle falls down to the mean value of about 72°. This decline is due to the formation of continuous Ag layer. The annealed samples exhibit entirely different dependence of CA on the sputtering time. The annealing of ultrathin Ag layers results in slight decrease of CA for sputtering times of 10 to 30 s. The initial drop is followed by a gradual increase of CA up to the value close to that of pristine PTFE. For thicker layers (sputtering times > 80 s), the CA remains practically constant, reflecting the fact that the post-deposition annealing leads to the coalescence of the Ag atoms into discrete islands (see Figure 2 and Table 1) and partial uncovering of the PTFE surface. Anomalous drop of contact angle at the initial stage of deposition is probably due to the disposition of silver to react with oxygen from ambient atmosphere (see, e.g., [20]). This phenomenon is particularly pronounced in tiny Ag structures [21]. Oxygen-rich compounds increase the sample wettability (see also Table 1; Ag/O ratio becomes lower for thin annealed layers).

**Table 1:** XPS elemental analysis of the Ag/PTFE composites

**Samples**	**Sputtering time (s)**	**Elemental composition (at.%)**			
		**Ag**	**O**	**F**	**C**
As-sputtered	20	11.7	2.8	37.3	48.2
	100	28.7	8.5	7.9	54.8
	200	29.9	15.3	-	54.8
Relaxed	20	11.0	6.6	30.1	52.3
	100	23.6	6.0	21.1	49.3
	200	25.0	10.2	2.0	62.8
Annealed	20	-	-	66.0	34.0
	100	2.5	0.9	57.7	39.0
	200	4.4	0.7	59.6	35.3

### UV–vis spectroscopy

**Figure 3:**
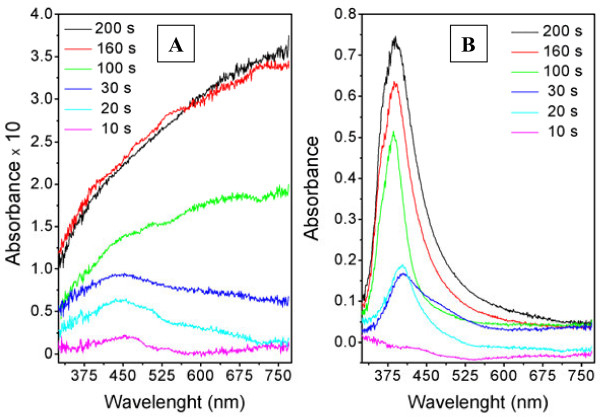
**UV–vis absorption spectra of silver-coated PTFE.** Relaxed **(A)** and annealed **(B)** samples sputtered for different times.

UV–vis absorption spectra of relaxed (A) and annealed (B) samples are shown in Figure 3. As expected, the absorbance increases with increasing deposition time as the Ag layer becomes thicker. The spectra of the annealed samples exhibit distinctive narrow absorption peak at about 400 nm, corresponding to the surface plasmon resonance (SPR) in silver nanostructures. It is well known that the position and shape of the SPR peak is closely related to the nanostructure shape and to the surrounding medium [22,23]. The appearance of absorption peak after annealing indicates the formation of discontinuous Ag clusters of hummock-like shape (see Figure 2) homogeneously distributed over the PTFE surface [24]. The absorption band corresponding to the bounded plasma resonance in the metal nanostructures is slightly shifted to longer wavelengths when the cluster density increases. Moreover, as the silver layer becomes thicker, the absorption band broadens due to wider distribution of the cluster size. The spectra of the as-deposited samples (Figure 3A) with deposition times below 30 s possess only weak SPR peak. In this case, the SPR peak is widespread and hardly identifiable because of insufficient separation of fundamental building blocks (clusters) of silver layer in the initial stage of the layer growth, where the formation of discontinuous but interconnected Ag coating is expected [19].

### Chemical composition

Besides the wettability, the chemical composition of the sample surface plays essential role in material biocompatibility [25,26]. Moreover, the elemental composition is closely linked to the wettability. The results from the XPS elemental analysis of the Ag/PTFE composites are summarized in Table 1. The average information depth of the present XPS measurement is limited to approximately 8 to 10 atomic surface layers. One can see that with ongoing deposition, the concentration of silver increases, while the fluorine content decreases and becomes undetectable on the sample sputtered for 200 s. The decrease is due to the increasing masking effect of the growing Ag layer which at last becomes homogeneous and continuous. On the other hand, with decreasing thickness of Ag layer, its masking effect gradually declines, e.g., because of the appearance of cracks and discontinuities in the layer, and the chemical structure of the underlying PTFE becomes visible in the XPS spectra. For the sputtering time of 20 s, the measured fluorine concentration of 37.3 at.% is close to that of the pristine PTFE. The F/C ratio of silver-sputtered samples is markedly different from that of the pristine PTFE (F/C = 2:1) and may be due to the ability of silver to attract hydrocarbon contaminants from ambient atmosphere [27]. The thicker the sputtered layer, the lower the F/C ratio. This effect is most pronounced in the case of the thickest Ag layer (200-s sputtering time), where fluorine is not detected because of the masking effect of the silver coating. However, the concentration of carbon is still notable (54 at.%) in this case. The origin of carbon may completely be attributed to the contamination with hydrocarbons and other carbon-rich compounds from ambient atmosphere.

XPS data (Table 1) also elucidate the processes in the course of the sample relaxation. During the 14 days of relaxation, the surface chemical composition changes significantly. A gradual decrease of the detected silver content, compared to that of the as-sputtered samples, occurs as a consequence of the tendency to minimize surface energy at the metal-polymer interface. This phenomenon has been frequently observed especially in the case of plasma-treated polymers, where oxygen-containing groups reorient towards polymer volume in order to reduce surface energy in the contact with ambient atmosphere [28]. Thus, the relaxation leads to segregation on the metal-polymer interface and boarding of cracks in the silver coating (Table 1, increase of fluorine content). This process favorably affects the surface wettability which finally stabilizes at a constant level (Figure 1). However, there are other concurrent processes that make the simple and straightforward explanation of the observed phenomena difficult (e.g., anomalous decrease of fluorine content for deposition time of 20 s, Table 1). This may particularly be caused by random, uncontrollable adsorption of hydrocarbons from ambient atmosphere during the relaxation process (see decrease of oxygen content at 100 and 200 s deposition times, Table 1). Since the changes of the surface properties (chemical composition and wettability) during the sample relaxation lead to the stabilization of the sample properties (Figure 1) and, in this way, to the improvement of reproducibility of further experiments on biocompatibility, these were performed on relaxed samples only.

Dramatic change in the surface chemistry occurs after the annealing (Table 1). Sharp drop in silver concentration for the samples sputtered for 100 and 200 s is caused by intensive coalescence of the Ag atoms into island-like formations (also Figure 2). This phenomenon is most pronounced for the sample sputtered for 20 s, in which no Ag is detected by the XPS method. With proceeding Ag coalescence, the F concentration increases dramatically as the original PTFE surface becomes uncovered, and simultaneously the measured F/O ratio approaches the value of pristine PTFE (F/O = 2:1). The lack of oxygen after the annealing may be attributed to the well-described effect of desorption of oxygen-rich contaminated product and reduction of oxidized silver [27].

### Surface morphology and roughness

Surface roughness and morphology of the substrates play a crucial role in adhesion and proliferation of cells [29,30]. AFM images of pristine, relaxed, and annealed silver-coated PTFE are shown in Figure 2 together with the corresponding values of surface roughness *R*_*a*_ (Table 2). For the sake of comparison, appropriate vertical scales were chosen for the particular images. The surface roughness of the relaxed Ag films decreases with increasing deposition time (Table 2), the decrease reflecting the layer growth mechanism [31]. During the initial stage of the layer growth, isolated silver islands (separated clusters) are formed, and the surface roughness increases compared to that of the pristine polymer. Longer deposition leads to the formation of interconnections between clusters, and the deposited layer becomes more homogeneous and uniform (see Table 1). This process is accompanied by gradual decrease of the surface roughness. Subsequent annealing results in pronounced change in the surface morphology. Annealing leads to silver coalescence and formation of hummock-like structures which are easily identifiable in the AFM images of samples which are Ag coated for different deposition times (Figure 2 annealed). This coalescence is due to the accelerated diffusion of Ag atoms at elevated temperature, and the formerly continuous Ag layer transforms into an island-like structure. The dimension of such structures is a function of the thickness of the Ag layer prior to annealing. The decomposition of the dense film into particles and clusters, known as solid-state dewetting [32], is driven by the minimization of surface energy. It should be noted that metals (e.g., gold) in the form of nanosized structures (rods, disks, and clusters) melt at lower temperatures than those in bulk materials. Those melting temperatures fall down to values between 300°C and 400°C, depending on the size and shape of the nanostructures [33,34].

**Table 2:** Mean surface roughness of pristine and Ag-coated PTFE for relaxed and annealed samples

**Sample**	**Sputtering time (s)**			
	**0**	**20**	**100**	**200**
Relaxed	27.5	36.5	27.3	22.6
Annealed	33.5	26.3	25.0	27.4

### Cell adhesion and proliferation

The adhesion and proliferation of VSMCs from the rat aorta were studied *in vitro* on the as-sputtered and annealed samples, both relaxed for 14 days. Cell adhesion is the first stage of cell-material interaction and occurs during the first 24 h from cell seeding. This process leads to the anchoring of the cells through specific binding interactions for a particular surface. Adhesion stage is controlled by the current state of the substrate surface. The second phase of the cell interaction is so called lag phase. It is the time required for cells to adapt to the new environment, and it takes approximately 24 to 48 h. After overcoming this stage, the cells can start to growth, spread, and proliferate.

**Figure 4:**
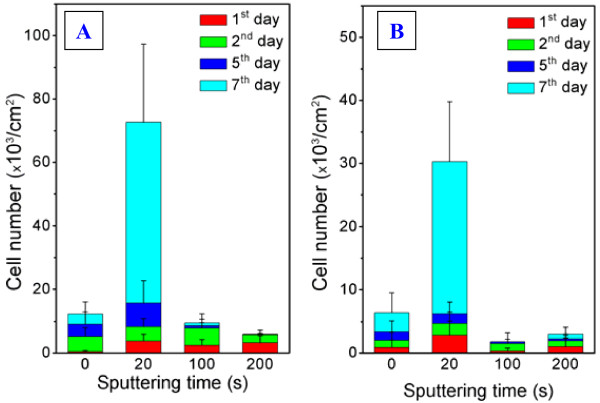
**The number of VSMC dependence on silver sputtering time.** The dependence of number of VSMCs on silver sputtering time for as-sputtered **(A)** and annealed **(B)** samples for different cultivation periods (first, second, fifth, and seventh days).

The degree of cell adhesion was determined as the number of cells found on the sample surface after 24 h from seeding. The dependence of the adhered VSMCs on the Ag sputtering time is shown in Figure 4A,B for relaxed and annealed samples. For comparison, the result for pristine PTFE (sputtering time 0 s) is also shown. From Figure 4A (as sputtered and relaxed samples) it is obvious that the presence of Ag coating has a positive effect on cell adhesion. The number of VSMCs found on the Ag-coated samples was comparable (3,150 ± 480 cells cm^−2^) for different sputtering times, whereas the adhesion on pristine PTFE was found to be very low (490 ± 280 cells cm^−2^). This result is rather unexpected since it is known that in general, the presence of nanosized Ag on tissue carriers has a negative effect on cell growth. In the case of the annealed samples (see Figure 4B), the situation is rather different. The highest increase of the adhered cells (2,830 cells cm^−2^) was observed on the sample sputtered for 20 s, while the cell adhesion on pristine PTFE and the samples Ag sputtered for longer deposition times (100 and 200 s) was minimal (Figure 4B). It is probably due to both lower wettability (caused by desorption of oxygen-rich compounds during annealing) and higher roughness of the samples.

Proliferation was determined as the number of VSMCs found on the samples after 2, 5, and 7 days from seeding (see Figure 4). The most significant changes were observed after the seventh day of cultivation. On the samples deposited for 20 s, a high cell number was found (72,650 ± 24,700 cells cm^−2^ for as-deposited and 29,300 ± 19,500 cells cm^−2^ for annealed samples). Higher proliferation on these samples occurred, owing to the formation of discontinuous metal layer and the favorable combination of the two factors, surface roughness and wettability. This result confirms the well-known fact that biocompatibility depends not only on the chemical composition but also on the surface properties such as the aforementioned wettability and roughness [35]. The low contact angle (high wettability), presence of oxygen in the surface layer, and rough surface of the substrate are prerequisites for successful VSMC adhesion. Thus, the difference in the number of proliferated cells between annealed and relaxed samples can be attributed to the different elemental compositions of the surface layer and resulting different wettability. From Figure 4A,B, it is evident that the cell proliferation on the other samples, sputtered for longer times, is very low. Sputtering for longer times (100 and 200 s), which leads to the formation of homogenous and continuous metal coverage, has a negative effect on cell interaction from the long-term point of view.

**Figure 5:**
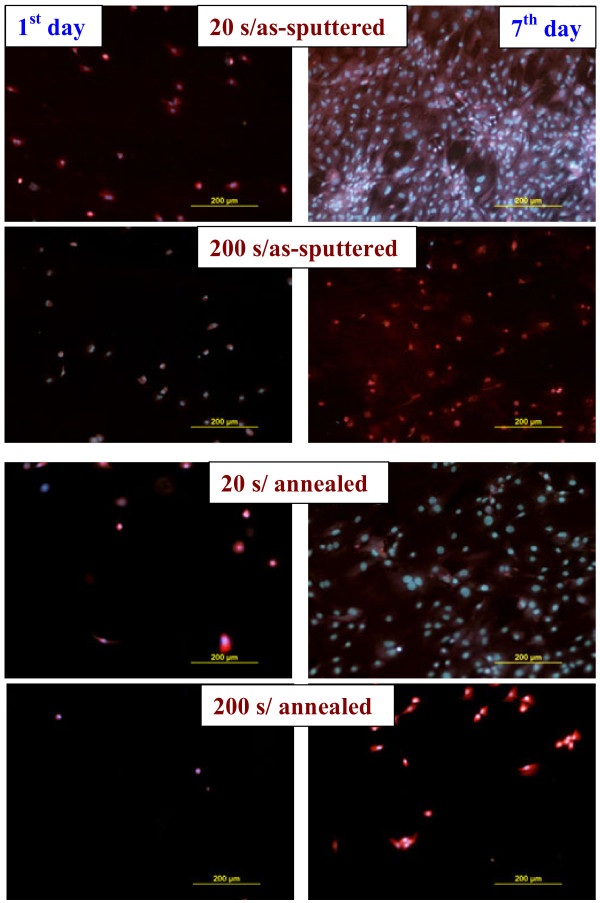
**Photographs of adhered and proliferated VSMCs.** Photographs of VSMCs adhered (first day) and proliferated (seventh day) on Ag-coated PTFE with different deposition times (20 and 200 s) for as-sputtered and annealed samples.

The above results are illustrated on the photographs of the adhered (first day from seeding) and proliferated (seventh day from seeding) cells on the relaxed and annealed samples (Figure 5). The cells cultivated for 24 h are equally distributed on the surface. The cells on the samples that are as-sputtered for 20 s and those on subsequently annealed samples start spreading, and their adhesion increases; however, the cells on the samples sputtered for 200 s and coated completely with silver stay small and round shaped. After 7 days from the seeding, the cells on the samples sputtered for 20 s are numerous and evenly distributed over the sample surface. The cell proliferation on the samples sputtered for 200 s is much worse. In the case of the as-sputtered layer, the silver forms homogenous coverage, completely shading the original polymer surface. After annealing of the thicker Ag layer, a dramatic coalescence of silver into distinctive hummock-like structures takes place, the latter being high enough to prevent a contact between polymer substrate and adhered cells.

## Conclusions

The properties of silver layers sputtered on PTFE for different times and their changes under annealing were studied by different methods. The biocompatibility of the samples prepared under different conditions was examined *in vitro* experiments with vascular smooth muscle cells. Relations between physicochemical properties of silver layers and their biocompatibility were found. Coating with silver leads to an increase of surface wettability, which is further affected by oxidized structures adsorbed by the sample surface. With the increasing thickness of the silver layer, an increase of the oxygen concentration is also observed which is explained by high affinity of silver to oxygen and oxidized structures. Post-deposition annealing leads to the dramatic change in morphology of the deposited silver layer and silver coalescence. Formerly, continuous or semicontinuous Ag layers are transformed into discontinuous ones, consisting of discrete hummock-like structures. In this way, the surface of PTFE may be partly uncovered by annealing. UV–vis absorption increases with increasing deposition time as the Ag layer becomes thicker. The UV–vis spectra of the annealed samples exhibit distinctive narrow absorption peak at about 400 nm, corresponding to the SPR in the silver nanostructures. The detailed characterization of Ag/PTFE composites, prepared under different conditions, was a prerequisite for the next experiments on their biocompatibility. The most important contribution of this work is the finding that the silver nanostructures, which are generally known for their inhibitory properties towards broad spectrum of bacterial strains and cells, under such specific conditions conform to cell cultures cultivated on PTFE support coated with those nanostructures. Best biocompatibility, cell adhesion, and proliferation were exhibited by the PTFE samples Ag sputtered for 20 s. Post-deposition annealing does not improve the sample biocompatibility. Increased biocompatibility of the samples coated with thin Ag layer is explained by favorable combination of the sample surface morphology and higher wettability. The biocompatibility of the samples sputtered for longer times and coated with thicker Ag layer is miserable. Last but not least, the results obtained by different diagnostic techniques on Ag/PTFE composites are of importance for better understanding of the growth mechanism of metal layer on polymer substrates and their behavior under annealing.

## Competing interests

The authors declare that they have no competing interests.

## Authors’ contributions

JS conceived of the study, carried out the thickness and AFM measurements. He designed and drafted the study. MP carried out and evaluated the contact angle and UV–vis measurements. NSK performed the cell adhesion and proliferation measurements together with its evaluation. ZK participated in the determination of the chemical composition. VS participated in the design of the study and its coordination. All authors read and approved the final manuscript.
